# Ravulizumab in the Management of Refractory Myasthenic Crisis: Clinical and Ventilatory Evidence of Early Recovery—A Case Report

**DOI:** 10.1155/crnm/1381459

**Published:** 2025-11-29

**Authors:** Manlio Sgarzi, Paolo Paone, Paolo Gritti, Emanuela Agazzi, Giorgia Camera, Dario Alimonti

**Affiliations:** ^1^Department of Neurosciences, Neurology Unit, Papa Giovanni XXIII Hospital 24127, Bergamo, Italy; ^2^Emergency Department, Unit of Anesthesia and Intensive Care 2, Papa Giovanni XXIII Hospital 24127, Bergamo, Italy

## Abstract

**Introduction:**

Generalized myasthenia gravis (gMG) is an autoimmune disorder impairing neuromuscular transmission, most commonly through anti-AChR antibodies that activate the complement cascade. This can lead to severe complications such as myasthenic crisis (MC), which often requires intensive care. While plasma exchange (PLEX) and intravenous immunoglobulins (IVIG) are standard first-line therapies, approximately 30% of patients may show suboptimal response. Ravulizumab, a long-acting C5 complement inhibitor, has been approved for anti-AChR-positive gMG, but data on its use in MC remain limited.

**Case Presentation:**

We report a 62-year-old male with late-onset, anti-AChR-positive gMG who presented with refractory MC, unresponsive to five PLEX sessions and IVIG. After infectious disease evaluation, meningococcal prophylaxis, and antibiotic coverage, a single intravenous loading dose of ravulizumab (2700 mg) was administered on ICU Day 9.

**Clinical Response:**

Marked clinical improvement was observed within 48 h, including reduction in ventilatory support (pressure support decreased from 16 to 6 cmH_2_O over five days), improved cough, secretion management, and eventual successful extubation on Day 17. By Day 21, the patient resumed oral feeding and was transferred out of ICU with stable respiratory function and neurological improvement.

**Conclusion:**

This case suggests that ravulizumab may provide rapid and sustained benefit in anti-AChR-positive patients experiencing refractory MC. Complement inhibition led to early ventilatory and neuromuscular recovery despite prior treatment failure. These findings support further investigation of ravulizumab as rescue therapy in acute, treatment-resistant gMG exacerbations.

## 1. Introduction

Generalized myasthenia gravis (gMG) is an autoimmune disorder impairing neuromuscular transmission, primarily due to antibodies against nicotinic acetylcholine receptors (AChRs) but also targeting MuSK and LRP4. Anti-AChR antibodies activate the complement system, leading to neuromuscular junction inflammation—a key driver of gMG pathogenesis [1]. Clinically, gMG causes progressive muscle weakness and fatigue. Myasthenic crisis (MC) is a severe complication requiring ventilatory support, enteral nutrition, and Intensive Care Unit (ICU) care [2]. First-line therapies include IVIG or plasma exchange (PLEX), effective in ∼60–70% of severe cases, typically within 3–5 days for IVIG or after 3 PLEX sessions [3, 4]. Ravulizumab, a humanized monoclonal antibody targeting complement C5, was recently approved for AChR-positive gMG [5]. We present a case of an AChR-positive patient in refractory MC, unresponsive to PLEX, who showed rapid ventilator weaning and resolution of bulbar symptoms following ravulizumab initiation.

## 2. Case Presentation

We report a 62-year-old man diagnosed in 2019 with late-onset anti-AChR + myasthenia gravis at 57. Initially, ocular (MGFA Class I) symptoms progressed to gMG (Class IIb) with bulbar and limb weakness. Comorbidities included hypertension (olmesartan 20 mg/die) and Grade I obesity (BMI ∼35). No thymectomy was performed. Since February 2025, the patient's condition had progressively worsened despite ongoing immunosuppressive therapy and a course of five PLEX sessions, which were preferred over intravenous immunoglobulin (IVIG) infusions, as the latter had previously proven ineffective. On April 9, 2025, the patient underwent neurological evaluation at ASST Papa Giovanni XXIII due to worsening generalized weakness and the onset of bulbar symptoms. On that occasion, the corticosteroid dose was increased from 30 mg/day to 62.5 mg/day, azathioprine was initiated at 100 mg/day, and pyridostigmine was added at a dose of 180 mg in the extended-release formulation, combined with four daily doses of 60 mg. On April 16, 2025, at 4:31 p.m., the patient presented to the Emergency Department of ASST Papa Giovanni XXIII because of further clinical deterioration, with dysphagia and dyspnea even at moderate exertion. Following neurological assessment, the patient was admitted to the sub-Intensive Care Unit (sICU), where at 12:11 a.m. on April 17, 2025 (Day 1), a nasogastric tube was placed due to severe dysphagia and neck flexor weakness. Five PLEX sessions were scheduled, with no changes made to the ongoing pharmacological therapy. The first two PLEX sessions were performed on the mornings of April 17 and 18, replacing approximately 3150 mL of plasma with an equivalent volume of 4% albumin solution. On the second day, the patient experienced a syncopal episode with bradycardia (28 bpm), which was managed conservatively. Because of diarrhea, the pyridostigmine dose was reduced to 30 mg four times daily (QID) plus 180 mg in the extended-release formulation. On the third day, at 9:21 a.m., the patient exhibited marked tongue weakness with intermittently incomprehensible nasal speech, orbicularis oculi weakness, a respiratory rate of 30 breaths per minute, hypoxemia, and hypocapnia. Due to respiratory compromise, the patient was transferred to the ICU. At 6:48 p.m. on April 19 (Day 3), the patient was intubated via direct laryngoscopy, while maintaining the ongoing medical therapy. On Days 4, 5, and 6, three additional PLEX sessions were performed using the same protocol as before, without any modification of pharmacological treatment. After the final PLEX session, neurological reassessment showed stability and mild improvement. On the morning of Day 7, following antibiotic prophylaxis, an extubation attempt was performed. On Day 8, due to ineffective cough and severe bulbar dysfunction, IVIG therapy was initiated at a total dose of 2 g/kg, and the patient was reintubated. The following morning, in the absence of any improvement in bulbar symptoms and considering the patient's refractoriness to first-line therapies, persistent clinical instability, prior lack of benefit from IVIG infusions, and the increasing risk of requiring tracheostomy, the medical team jointly decided to initiate complement inhibition therapy without further delay. Antibiotic prophylaxis and the required meningococcal vaccinations were planned and administered on the same day. On Day 9, the patient continued all ongoing medications without changes, and at 10:04 a.m. on April 25, 2025 (Day 9), the first dose of ravulizumab (2700 mg IV) was administered.

## 3. Treatment With Ravulizumab

### 3.1. Clinical Response

Day 10: The patient remained on mechanical ventilation.

Day 11: The patient was awake, cooperative, with good peripheral strength; ventilated on minimal PSV, optimal gas exchange.

Day 12: The patient completed meningococcal ACWY and Hib vaccinations. Secretions were manageable; effective cough.

Days 13–15: Progressive respiratory and neurological recovery, improved swallowing and secretion control, and labs normalized.

Day 16: Brief weaning from ventilation, improved strength, and reduced sialorrhea.

Day 17: Successful extubation with stable gas exchange and spontaneous breathing.

Day 21: Nasogastric tube removed after positive speech evaluation, improved swallowing and speech, and transferred from ICU to Neurology in stable condition with marked MG improvement.

Day 23: Fully independent breathing, stable blood gases, reduced fatigue, and improved proximal strength. Clinical response remained stable on short-term follow-up.

### 3.2. Trend of Blood Gas and Ventilatory Parameters

**Figure 1:**
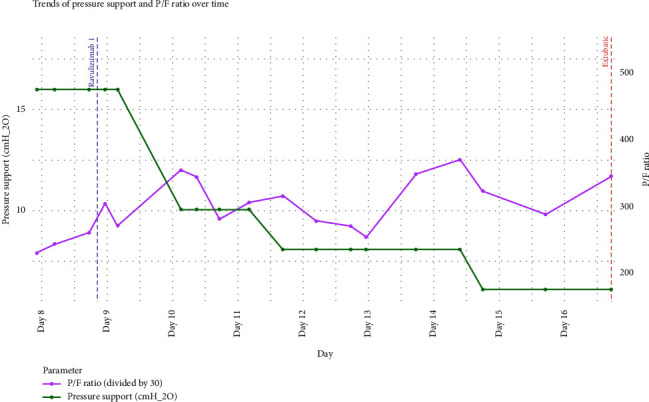
Trend of key ventilatory parameters in the ICU before and after the first infusion of ravulizumab.

**Figure 2:**
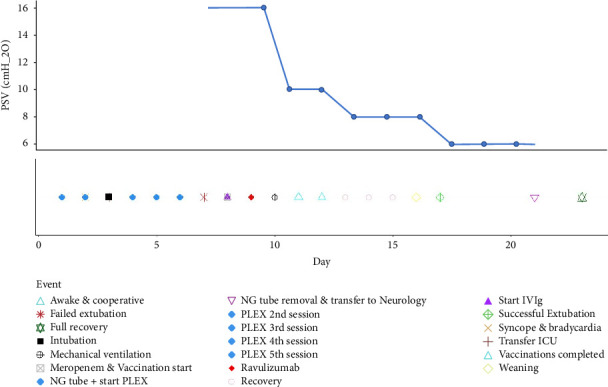
Trend of pressure support ventilation (PSV) levels and major clinical events during the patient's Intensive Care Unit (ICU) stay.

After the first ravulizumab dose, we observed a progressive improvement in respiratory function, supported both by blood gas analysis and ventilator parameters. Pressure support (P. Supp) decreased from 16 to 6 cmH_2_O over five days. Specifically, the initial decrease from 16 to 10 cmH_2_O occurred the day after the loading dose of ravulizumab, while blood gas parameters remained stable. The initial drop from 16 to 10 cmH_2_O occurred the day after dosing, with stable blood gases (Figures 1 and 2). This gradual P. Supp reduction, without blood gas worsening, suggests decreased respiratory effort and possible neuromuscular respiratory recovery (Table 1).

## 4. Discussion

Ravulizumab, a next-generation complement C5 inhibitor derived from eculizumab, has a much longer half-life, allowing dosing every 8 weeks after an initial loading dose. This ensures comparable efficacy, reduced hospital burden, and stable serum levels [6]. Its efficacy in anti-AChR + gMG is established, but its role in MC remains under study. Our case supports rapid, meaningful complement inhibition in acute settings, with patient improvement within 48–72 h and neuromuscular stabilization over 7–10 days, consistent with refractory MC reports [9]. Ravulizumab rapidly saturates free C5 (< 0.5 μg/mL) and maintains therapeutic levels > 8 weeks. Target concentrations (> 175 μg/mL) are reached within 30 min postinfusion, consistent across weights [7], enabling sustained complement suppression—a key in MG pathophysiology. Reports, including Konen et al.'s study [8], show early, sustained neuromuscular gains in refractory cases. The progressive improvement in respiratory parameters observed after ravulizumab administration appears clinically relevant and temporally related to the start of treatment. In particular, the reduction of pressure support (P. Supp) from 16 to 6 cmH_2_O in only five days, accompanied by stabilization of blood gas values, suggests increased ventilatory efficiency and early recovery of respiratory muscle strength. These data are consistent with the hypothesis of a favorable effect of ravulizumab on the complement cascade, which may help reduce immune-mediated neuromuscular damage. In particular, stable lactate levels and the absence of changes in FiO_2_ strengthen the idea that ventilatory improvement is linked to early recovery of respiratory muscle strength [10]. This effect deserves further investigation in prospective controlled studies to better define the role of complement blockade in respiratory function recovery in selected clinical settings. The rationale is particularly strong in AChR + patients with documented complement activation and in cases of first-line therapy failure. Limitations in this case report include short follow-up, potential confounding from concurrent therapies (PLEX, antibiotics, and corticosteroids), and lack of systematic MG-ADL or QMG scoring, limiting objective comparison.

## Figures and Tables

**Table 1:** Respiratory and metabolic parameters monitored daily in patient.

Day	Day 8	Day 8	Day 9	Day 9	Day 9	Day 10	Day 10	Day 11	Day 11	Day 12	Day 12	Day 13	Day 13	Day 14	Day 14	Day 15	Day 16	Day 17
Hours	12:00	18:14	07:14	13:11	17:41	16:57	22:40	07:12	18:00	06:24	18:38	07:14	13:08	07:15	23:31	07:55	06:47	07:03
P. Supp (cmHO2)	16	16	16	16	16	10	10	10	10	8	8	8	8	8	8	6	6	6
Ph	7.45	7.4	7.43	7.47	7.46	7.42	7.47	7.48	7.45	7.45	7.46	7.45	7.43	7.43	7.42	7.42	7.45	7.44
pCO_2_ (mmHg)	37	36	42	35	37	41	37	36	37	41	35	41	39	43	45	44	40	43
pO_2_ (mmHg)	94	99	106	123	110	143	139	114	124	128	113	110	103	141	150	131	117	140
O_2_Hb %	97.3	96.9	97.7	97.8	97.4	97.3	98.2	97.7	98	97.6	97.9	97.5	97.5	97.7	98.1	98.2	98.1	97.8
Na^+^: (mmol/L)	133	131	132	131	133	133	132	133	131	133	133	133	132	135	134	134	134	134
K^+^: (mmol/L)	4.5	4.5	3.8	4.5	4.6	4.4	4.2	3.5	4.5	3.7	4.2	3.4	4.2	4	3.9	3.3	3.4	3.8
AG (mmol/L)	9	9	5	7	7	10	6	7	9	5	10	6	9	8	7	8	8	8
Lactate (mmol/L)	0.7	2.1	1	1.4	1.9	2.1	2	1.2	2.5	1.1	1.5	1.1	1.9	1.7	2	1.2	1.1	1
FIO_2_%	40	40	40	40	40	40	40	40	40	40	40	40	40	40	40	40	40	40
P/F rate	235	248	265	308	275	358	348	285	310	320	283	275	258	353	375	328	293	350
TCO_2_ (mmol/L)	26.8	25	29.2	26.6	27.4	27.9	28	27.9	26.8	29.8	26	29.8	27.1	29.8	30.6	29.9	29	30.5

*Note:* P. Supp: pressure support, pH: arterial pH, pCO_2_: partial pressure of carbon dioxide, pO_2_: partial pressure of oxygen, O_2_Hb: oxyhemoglobin saturation, Na^+^: sodium, K^+^: potassium, AG: anion gap, lactate: blood lactate, FiO_2_: fraction of inspired oxygen, P/F ratio: PaO_2_/FiO_2_, and TCO_2_: total carbon dioxide.
